# Awareness of osteoporosis among 368 residents in China: a cross-sectional study

**DOI:** 10.1186/s12891-020-03217-1

**Published:** 2020-03-30

**Authors:** Kemal Sherefa Oumer, Yawen Liu, Qiong Yu, Fan Wu, Shuman Yang

**Affiliations:** Department of Epidemiology and Biostatistics, School of Public Health, Jilin University, 232-1163 Xinmin Street, Changchun, 130021 Jilin China

**Keywords:** Osteoporosis, Awareness, Knowledge, China

## Abstract

**Background:**

Studies on osteoporosis awareness among the general population in China are still limited. We examined the level of osteoporosis awareness among residents in China, determined the risk factors associated with a lower level of osteoporosis awareness, and assessed the sources of their knowledge about osteoporosis.

**Methods:**

We conducted a cross-sectional study among 368 general residents aged 30 years or older from 19 provinces during January–March 2018 in China. All participants were identified and interviewed face-to-face by medical students in Jilin University using a structured questionnaire. Osteoporosis awareness scores (percent of the correct answer) were determined across several domains, including definition, diagnosis, risk factors, and prevention of osteoporosis. We used multiple linear regression models to test the relationship between risk factors and overall awareness scores.

**Results:**

The mean age of included participants was 52.9 ± 10.2 years, and 53% of them were male. Osteoporosis awareness score for definition was 77.7%, diagnosis 49.6%, risk factors 49.2%, treatment 60.5%, and prevention 69.9%. The overall awareness score was 67.8%. Lower family income and education level were significantly associated with lower overall awareness score (all *p* < 0.05). Television or radio health program was reported to be their main source of knowledge about osteoporosis.

**Conclusion:**

The awareness level for osteoporosis in our study is moderate; lower family income and education level were risk factors for lower awareness. Television or radio health programs had the greatest contribution to osteoporosis awareness.

## Background

Osteoporosis characterized as low bone mass and micro-architectural deterioration is a major public health problem. It is estimated to affect 200 million women worldwide and cause more than 8.9 million fractures annually [1]. By 50 years of age, one in three women and one in five men will suffer a fracture in their remaining lifetime [2]. It is projected that by 2050, 50% of hip fractures will occur in Asia, with the majority occurring in China [3]. Osteoporosis affects almost 7.0 million Chinese over the age of 50 and causes about 687,000 hip fractures in China each year [4]. Improving osteoporosis awareness is useful for controlling this problem because a high level of osteoporosis awareness increases the possibility of early osteoporosis detection and promotes effective osteoporosis prevention and treatment [5, 6].

Although there are several Chinese studies that have examined osteoporosis awareness [7–12]. Studies on osteoporosis awareness among the general population in China are still limited. Also, no studies have determined the risk factors associated with lower levels of osteoporosis awareness and assessed the way of source of knowledge about osteoporosis. Therefore, we examined the level of osteoporosis awareness among residents in China, determined the risk factors associated with a lower level of osteoporosis awareness, and assessed the sources of their knowledge about osteoporosis.

## Methods

### Study setting and population

This cross-sectional survey was conducted from 19 provinces during January–March 2018 in China. The inclusion criteria for the survey participants were 1) aged 30 years or older at the interview; 2) able to give consent to participate in the study, and 3) capable of understanding and answering the questions. The participants were identified and interviewed face-to-face by medical students in Jilin University (Enrolment year: 2017; Specialty: Preventive Medicine) using a structured questionnaire. The participants for this survey included the relatives, friends, and neighbours of the students. Before conducting the survey, all medical students were trained to identify suitable participants and conducting the survey with a structured questionnaire in a face-to-face meeting room by related researchers. This study was approved by the Ethical Committee Board at the School of Public Health, Jilin University. Each participant also provided written informed consent to this study.

### Study measures

Socio-demographics (sex, age, body weight, height, residence, education level, and family annual income), lifestyle information (smoking, alcohol use), prior fracture and prior bone mineral density test were collected using a structured questionnaire. Height and weight were self-reported. Body mass index (BMI) was calculated as body weight (kg) divided by squaring of body height (m^2^). Educational level was classified as primary and below (Years of education: 6 and below), junior (Years of education: 7–9), senior (Years of education: 10–12) and undergraduate and postgraduate educations (Years of education: 13 and above). A residence was classified as urban, rural and suburban.

We assessed osteoporosis awareness level using the following domains, including by definition, diagnosis, signs/symptoms, treatment, complications, prognosis, causes, risk factors, and prevention of osteoporosis; the questionnaire about osteoporosis awareness was the same as previously published research [13]. The reliability and validity of the questionnaire was tested among 30 Chinese subjects before the formal survey. This questionnaire had good internal consistency (Cronbach’s α = 0.746). Under the factorial validity test, the related components explained a cumulative 60% of the variance in the awareness scores. The Kaiser-Meyer-Olkin measure of sampling adequacy and Bartlett’s test of sphericity both showed that the results were suitable for factor analysis. Besides the osteoporosis awareness questions, we also collected the sources of the participants acquiring their existing osteoporosis knowledge (e.g., newspapers and magazines, advertising leaflets, television or radio health program).

### Statistical analysis

We used descriptive statistics to describe the characteristics of the study population and the main variables. Continuous variables were shown as mean ± standard deviation (SD); categorical variables were shown as percentages.

Awareness scores were created by assigning a “1” to each correct response and a “0” to each incorrect or “unsure” response. Awareness scores for all questions were summed for a possible range of 0 to 29; higher scores suggest greater awareness. The domain scores were an average of the percentages of correct answers to all questions under each domain.

To test the relationship between risk factors and overall awareness scores, we used multiple linear regression models. Covariates included sex, age, body mass index, residence, educational level, family annual income, prior bone mineral density test, prior fracture, smoking, and alcohol use.

All statistical analyses were performed by using SPSS software (version: 25.0; SPSS Inc., Chicago, IL).

## Results

In total, we collected 372 questionnaires; each student returned an average of six survey questionnaires. Among 372 questionnaires, four of them were excluded because of missing all data on osteoporosis awareness questions. We finally included 368 valid questionnaires in our analysis (Table 1). The mean age of included participants was 52.9 ± 10.2 years; 53% were males; the average BMI was 23.7 ± 3.3 kg/m^2^. Approximately, 18.5 and 36% of participants reported being current smokers and alcohol users, respectively.

**Table 1:** Characteristics of study (*N* = 368)

Characteristics	Values
Male (n, %)	195 (53)
Age group (years)	
30–49 (n, %)	208 (56.5)
50–59 (n, %)	76 (20.7)
60–69 (n, %)	55 (14.9)
Above 70 (n, %)	29 (7.9)
Body mass index (kg/m^2^)	23.7 (3.3)
Residence	
Urban (n, %)	166 (45.1)
Rural (n, %)	113 (30.7)
Suburban (n, %)	89 (24.2)
Education	
Primary and below (n, %)	69 (18.8)
Junior (n, %)	113 (30.7)
Senior (n, %)	84 (22.8)
Graduate (n, %)	102 (27.7)
Family annual income (US dollar)	
< 1400 (n, %)	41 (11.1)
1400–6999 (n, %)	128 (34.8)
7000–13,999 (n, %)	97 (26.4)
≥ 14,000 (n, %)	102 (27.7)
Prior bone mineral density test (n, %)	68 (18.5)
Prior fracture (n, %)	48 (13)
Smoker	
Current (n, %)	68 (18.5)
Past (n, %)	45 (12.2)
Never (n, %)	255 (69.3)
Alcohol user (n, %)	133 (36.1)

Values are means (SD), unless otherwise specified

### Osteoporosis awareness level

Table 2 presents descriptive data about osteoporosis awareness scores by domain. Osteoporosis awareness score for definition was 77.7%, diagnosis 49.6%, risk factors 49.2%, treatment 60.5%, and prevention 69.9%. The overall awareness score was 67.8%.

**Table 2:** Frequencies and percents of correct response by domain (*N* = 368)

Domain	Domain score (%) (Correct response: T = True, F = False)	Correct response, N (%)
Definition of Osteoporosis	Osteoporosis is a condition of easy joint dislocation (F)	222 (60.3)
	Osteoporosis is a condition of low bone mineral density (T)	296 (80.4)
	Osteoporosis is a condition of high bone mineral density (F)	340 (92.4)
	Domain score = 77.7%	
Diagnosis of Osteoporosis	Osteoporosis is diagnosed using X-ray of the bone (T)	192 (52.2)
	Osteoporosis is diagnosed with physical exam (F)	173 (47.0)
	Domain score = 49.6%	
Common signs/ symptoms of osteoporosis	Headache is a common sign/symptom of osteoporosis (F)	310 (84.2)
	Frequent fractures is a common sign/symptom of osteoporosis (T)	312 (84.8)
	Mood change is a common sign/symptom of osteoporosis (F)	313 (85.1)
	Domain score = 84.7%	
Treatment of osteoporosis	Osteoporosis can be treated with calcium and vitamin D (T)	321 (87.2)
	Osteoporosis can be treated with surgical correction (F)	273 (74.2)
	Osteoporosis can be treated with hormone replacement (T)	74 (20.1)
	Domain score = 60.5%	
Complications of osteoporosis	Diabetes is a complication of osteoporosis (F)	313 (85.1)
	Hypertension is a complication of osteoporosis (F)	310 (84.2)
	Hip fracture is a complication of osteoporosis (T)	311 (84.5)
	Domain score = 84.6%	
Prognosis for Osteoporosis	Osteoporosis can lead to joint swelling and morning stiffness (F)	162 (44.0)
	Osteoporosis can lead to hip fractures and subsequent complications (T)	286 (77.7)
	Domain score = 60.8%	
Common causes of Osteoporosis	Overweight is a common cause of osteoporosis in women (F)	225 (61.1)
	Lack of estrogen is a common cause of osteoporosis in women (T)	156 (42.4)
	High protein diet is a common cause of osteoporosis in women (F)	263 (71.5)
	Domain score = 58.3%	
Risk factors for Osteoporosis	Low rice intake is a risk factor for osteoporosis in women (F)	233 (63.3)
	Post menopause is a risk factor for osteoporosis in women (T)	158 (42.9)
	Smoking is a risk factor for osteoporosis (T)	152 (41.3)
	Domain score = 49.2%	
Risk of osteoporosis over a woman’s lifetime	Women are at highest risk for osteoporosis at puberty (F)	315 (85.6)
	Women are at highest risk for osteoporosis during their childbearing ages (F)	244 (66.3)
	Women are at highest risk for osteoporosis after menopause (T)	248 (67.4)
	Domain score = 73.1%	
Prevention of Osteoporosis	Moderate physical exercise can reduce the risk of osteoporosis (T)	272 (73.9)
	Increased rice consumption can reduce the risk of developing osteoporosis (F)	276 (75.0)
	A diet rich in calcium and vitamin D can reduce the risk of developing osteoporosis (T)	306 (83.2)
	Cigarette smoking cessation can reduce the risk of developing osteoporosis (T)	175 (47.6)
	Domain score = 69.9%	

Table 3 indicates that lower family income and education level were significantly associated with lower overall awareness scores (all *p* < 0.05). However, all the other variables were not significantly associated with overall scores of osteoporosis awareness.

**Table 3:** The associations between risk factors and overall awareness score in the multivariable linear regression models

Variables	*β*	*SE*	*P* value
Male vs Female	−0.598	0.464	0.198
Age in year	0.008	0.019	0.676
Body mass index (kg/m^2^)	0.108	0.057	0.060
Residence			
Urban vs Rural	0.651	0.543	0.231
Suburban vs Rural	−0.292	0.547	0.595
Education level			
Junior vs Primary and below	1.551	0.568	0.007
Senior vs Primary and below	2.333	0.644	0.000
Graduate vs Primary and below	1.857	0.693	0.008
Family annual income (US dollar)			
< 1400 vs ≥14,000	−1.171	0.549	0.039
1400–6999 vs ≥14,000	−1.243	0.537	0.021
7000–13,999 vs ≥14,000	−1.136	0.532	0.033
Prior BMD test vs No prior BMD test	0.555	0.498	0.266
Prior fracture vs No prior fracture	−0.850	0.560	0.130
Smoker			
Current vs Never	−1.067	.703	0.130
Past vs Never	−0.581	0.659	0.378
Alcohol user vs No alcohol user	1.021	0.535	0.057

*Abbreviations*: *β* regression coefficient, *SE* standard error, *BMD* bone mineral density

*P* significant at ≤0.05

With regard to sources of knowledge about osteoporosis, television or radio health program was reported to be their main source of knowledge about osteoporosis (Table 4).

**Table 4:** Percent for each source for obtaining existing osteoporosis knowledge

Source	Percent
Newspapers and magazines	29.1
Advertising leaflets	11.1
Community public paper material	15.2
Community health knowledge lecture	15.8
Television or radio health program	55.7
Internet	22.6
Wechat group	29.1
Chatting with friends and family members	40.5
None	10.1

## Discussion

In China, osteoporosis is still a major public health problem with a growing aging population. Raising osteoporosis awareness among the general population is useful for osteoporosis prevention and treatment programs [14]. This study examined the level of osteoporosis awareness among Chinese residents, determined the risk factors associated with a lower level of osteoporosis awareness, and assessed the sources of their knowledge about osteoporosis. We restricted participants aged 30 years or older because bone loss normally begins at that age and assessing osteoporosis awareness for these people is useful for more effective osteoporosis prevention and treatment [15–17].

In this study, the overall osteoporosis awareness score was 67.8%; 83.3% of the respondents knew correctly aware of the definition of osteoporosis. These figures are higher than a study in Vietnam in which participants answered 49% of the knowledge questions correctly [13]. This discrepancy may be because our study participants have a higher education level than the Vietnamese participants (With graduated education: 27.7% vs. 19.4%, respectively). Because most of the other studies used different questionnaires for assessing osteoporosis awareness [18–20], we can only compare the osteoporosis definition of awareness between studies. The studies in South Australia, Canada, and Greece found that 62–96% of participants correctly aware of the osteoporosis definition. These results are comparable to our study.

Regardless of consistent and inconsistent results, our study found a low awareness score of the osteoporosis risk factors. In our study, one in every two participants did not know the risk factors and the diagnosis. This figure is much higher than a South American study, in which one in every ten participants did not know the osteoporosis risk factors [21]. This is likely attributed to the fact that the participants in the South American study have higher risk of osteoporosis than those in our study (Mean age: 63.0 vs. 52.9 years; percent for prior fracture: 49% vs. 13%, respectively), and higher osteoporosis risk has been suggested to be positively associated with osteoporosis awareness [12].

It is generally believed that osteoporosis awareness level is greater in females than in males. This is not consistent with our study, in which females and males have comparable osteoporosis awareness levels. In our study, males have a better education than females in our study (Percent with graduated education: 33.8% in males vs. 20.8% in females; *P* < 0.001); this may lead to similar osteoporosis awareness scores between sexes.

Our results showed a higher level of education was associated with better awareness of osteoporosis; this was also found in other studies [12, 22]. Individuals with better education may have a greater willingness to obtain osteoporosis-related knowledge.

In our study, 55.7% of participants reported television or radio health program as their main source of osteoporosis knowledge. The main source of knowledge was also a television for the Canadian, Singaporean, and American studies [20, 22, 23]. This provides an important and effective way of disseminating osteoporosis knowledge to the general population.

Our study has an advantage and two limitations. Although the questionnaire about osteoporosis awareness has been used in a previous study, we still validated the questionnaire prior to the formal survey. Our reliability and validity test results suggested the questionnaire is suitable for the present study. This study included only 368 participants; a small sample size has limitations to represent the general Chinese population. Lastly, due to cross-sectional design, we are unable to make causal inferences about the association between osteoporosis awareness score and related factors.

## Conclusions

The awareness level for osteoporosis in our study is moderate; lower family income and education level were risk factors for lower awareness. Television or radio health programs had the greatest contribution to osteoporosis awareness. These findings may be useful for osteoporosis prevention and treatment among the general Chinese population. For example, future osteoporosis educational programs may include more information on osteoporosis risk factors.

## Data Availability

The datasets used and/or analysed during the current study are available from the corresponding author on reasonable request.

## Abbreviations

BMD: Bone mineral density
BMI: Body mass index
SD: Standard deviation
SPSS: Statistical package for the social sciences
